# An experimental and numerical investigation of secondary char formation in hydrothermal carbonization: revealing morphological changes *via* hydrodynamics†

**DOI:** 10.1039/d4ra08995b

**Published:** 2025-04-23

**Authors:** Omar M. Abdeldayem, Capucine Dupont, David Ferras, Maria Kennedy

**Affiliations:** a Department of Water Supply, Sanitation and Environmental Engineering, IHE Delft Institute for Water Education Westvest 7 2611AX Delft The Netherlands o.m.h.m.abdeldayem@tudelft.nl o.abdeldayem@un-ihe.org omar.abdeldayem94@gmail.com; b Department of Water Management, Faculty of Civil Engineering and Geosciences, Delft University of Technology Stevinweg 1 2628 CN Delft The Netherlands

## Abstract

Hydrothermal carbonization (HTC) research has mainly focused on primary char production, with limited attention to secondary char, which is formed through polymerization and condensation of dissolved organic compounds in the liquid phase. This research aims to address this gap *via* an experimental investigation of the impact of stirring on the mass and carbon balance of HTC reaction products, surface functional groups, and surface morphology of secondary char, using fructose as a model compound. A 3D hydrodynamic simulation model was developed for a two-liter HTC stirred reactor. The experimental results indicated that stirring did not significantly influence the pH, mass, carbon balance, and surface functional groups of secondary char produced under the range of experimental conditions (180 °C, 10% biomass to water (B/W) ratio, and a residence time of 0–120 min) studied. Nonetheless, it was observed that a stirring rate of 200 rpm influenced the morphology and shape of the secondary char microspheres, leading to a significant increase in their size *i.e.*, from 1–2 μm in unstirred conditions compared with 70 μm at a stirring rate of 200 rpm. This increase in size was attributed to the aggregation of microspheres into irregular aggregates at stirring rates > 65 rpm and residence times > 1 h. The hydrodynamic model revealed that high turbulence of Re > 10^4^ and velocities > 0.17 m s^−1^ correlated with regions of secondary char formation, emphasizing their role in particle aggregation. Particle aggregation is significant above a stirring rate of 65 rpm, which corresponds to the onset of turbulent flow in the reactor. Finally, a mechanism is proposed, based on reactor hydrodynamics under stirred conditions, that explains secondary char deposition on the reactor walls and stirrer.

## Abstract 2

Experimental and hydrodynamic modeling of a two-liter HTC reactor using fructose as a model compound highlights the influence of stirring on secondary char morphology, size, and deposition.

## Graphical abstract

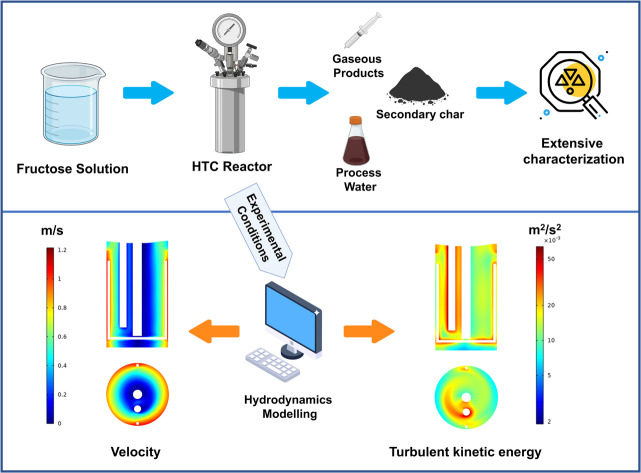

## 1. Introduction

The substantial volumes of wet biomass generated from various activities have recently gained interest as a sustainable solution for waste valorization. Hydrothermal carbonization (HTC) is considered a promising thermochemical process for wet biomass valorization (>50 wt%), providing a better alternative than conventional thermochemical processes, which require pre-drying, such as torrefaction, pyrolysis, and gasification.^1^ HTC operates at relatively low temperatures (180–250 °C), autogenic pressure (1–10 MPa), and typically residence times between 1 and 72 hours.^2^

During the HTC of biomass, water functions as a solvent and catalyst for multiple reactions, including hydrolysis, dehydration, decarboxylation, aromatization, condensation, and polymerization.^2^ The associated products of HTC can be categorized into three main phases: solid phase (primary char, secondary char, and carbon dots), liquid phase (organic compounds (mainly acids) and leached inorganics), and gaseous phase (mainly CO_2_).^3,4^ Primary char refers to the char produced from the solid–solid conversion of biomass and it has a similar structure and morphology to the original material. In contrast, secondary char refers to the char fraction produced from the polymerization and condensation reactions of dissolved organic compounds in the liquid phase (leached out during the HTC of raw biomass). Secondary char has a distinctive morphology consisting of microspheres. In previous studies, the terms secondary char, humins, and carbon microspheres have been used interchangeably and often with the same meaning.^3,5–9^ The terminological divergence comes from the fact that HTC is a relatively new field; hence, the terminology is not yet fully standardized. In this study, the term secondary char will be used. Lastly, carbon dots usually refer to carbon nanoparticles with particle sizes smaller than 10 nm having photoluminescence properties.^10^ However, the formation mechanism of carbon dots is not fully understood.^3^

Secondary char has gained considerable attention in the production of advanced carbon-based materials.^3^ Besides the complex HTC conversion pathways, which are still not fully comprehended, studying secondary char using heterogeneous biomass poses further challenges. The process includes chemical extraction methods to separate the secondary char from the main primary char.^11^ Hence, the extraction technique and solvent used could influence the properties of secondary char. Therefore, several studies have focused on examining secondary char from model compounds such as glucose, xylose and fructose.^3,11–13^ Indeed, when model compounds, such as simple sugars (glucose or fructose), are used in HTC, the experiment is conducted by adding (simple) sugars to water, forming a solution. This type of experiment only enables the formation of secondary char as a solid fraction from the polymerization and condensation reactions of organic acids, which are intermediate products in the HTC reactions of sugars.

Since the specificity of secondary char is a result of its surface properties, several studies have indicated the influence of process parameters, such as stirring, on surface morphology.^12,13^ However, to date, no detailed explanation has been proposed for the observed impact of stirring on the formation and morphology of secondary char.

Computational fluid dynamic (CFD) modeling is crucial in understanding and optimizing HTC. Hydrodynamic modeling, in particular, provides insights that can explain observations related to the impact of stirring on hydrochar and, more specifically, on secondary char. To date, most studies have focused on simulating hydrodynamics during primary char production; however, no study has investigated how hydrodynamics impact the production of secondary char. There are studies that have investigated the hydrodynamics of unstirred reactors^14,15^ using heterogeneous biomass or macroscopic solid particles.^16^ However, only one study conducted by Sangare *et al.*^17^ investigated the hydrodynamics of a stirred reactor using avocado stone as a feedstock and they indicated that a stirring rate of 550 rpm resulted in turbulent flow in the 300 mL reactor.

Based on the current knowledge gaps, the general objective of this work is to investigate the influence of stirring rate on the HTC of fructose, with a focus on understanding how stirring affects the physicochemical properties of the products and the formation mechanisms of secondary char. The specific objectives are to (i) investigate the influence of the stirring rate and flow regime on solid and carbon mass fractions as well as the properties of secondary char (proximate analysis, organic elemental composition, surface functional groups) using fructose as a model compound, (ii) describe the hydrodynamic conditions in the stirred HTC reactor by means of a 3D CFD model and (iii) explain the influence of stirring hydrodynamics on the formation and morphology of secondary char.

## 2. Methodology

### 2.1. Experimental

#### 2.1.1. Feedstock, HTC setup and procedure

This study employed d-fructose, sourced from corn with a purity exceeding 99%, obtained from Sigma-Aldrich.

The HTC experiments were conducted in a 2 L high-pressure reactor vessel (Parr series 4530-floor stand reactor). The full description of the reactor can be found in Abdeldayem *et al.*^18^ The fructose solution was introduced into the HTC vessel, occupying 70% (1.3 L) of its total volume. The reactor was purged for 3 minutes with grade-6 N_2_ gas to ensure an inert atmosphere. The reactor was heated for around 45 min to 180 °C leading to a heating rate of approximately 3 °C min^−1^. The reactor was then maintained at the operating temperature for the studied residence time and was cooled down in *ca.* 100 minutes using compressed air at the end of the experimental run. Collection of solid, liquid, and gaseous samples and their storage methods are performed according to the procedure described in Abdeldayem *et al.*^18^

#### 2.1.2. Experimental design

All HTC experiments were duplicated, as shown in the experimental design in Table 1. The experiments covered different ranges of residence time to investigate the kinetics of secondary char formation. A 180 °C temperature was chosen, as a previous study indicated that the solid mass fraction of secondary char increases significantly at 180 °C by changing residence time and above that temperature, the solid mass fraction stabilizes faster.^3^ In addition, the stirring rate was investigated at 0, 65 (only for morphology), and 200 rpm (clockwise direction) to unveil the influence of stirring on secondary char formation at laminar and turbulent flow conditions.^18^ Reynolds number (Re) was used to estimate the flow turbulence, as shown in eqn (1), for which the flow is considered laminar if Re < 10, and fully turbulent if Re > 10^4^:^17,19^1
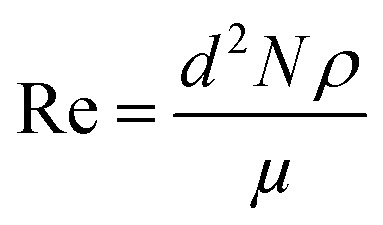
where *d* (m) is the diameter of the impeller, *N* (s^−1^) is the impeller's rotational frequency, *μ* (Pa s) is the dynamic viscosity and *ρ* (kg m^−3^) is the density.

**Table 1:** Experimental design

Sample name	Temperature (°C)	Residence time (min)	Stirring rate (rpm)	B/W ratio (%)
F180-0-200	180	0	200	10
F180-0-0	180	0	0	10
F180-15-200	180	15	200	10
F180-15-0	180	15	0	10
F180-60-200	180	60	200	10
F180-60-100	180	60	100	10
F180-60-30	180	60	30	10
F180-60-0	180	60	0	10
F180-120-200	180	120	200	10
F180-120-0	180	120	0	10

#### 2.1.3. Gas, liquid, and solid characterization

Gas chromatography was used to analyze the components in the gaseous products. The liquid products were analyzed using Shimadzu TOCv-cpn for dissolved organic carbon (DOC). The Modular Compact Rheometer: MCR 72/92 was used to measure the viscosity of the fructose solution, while the density of the fructose solution was determined by measuring the specific gravity using a hydrometer IN-032.

The elemental composition in C, H, N, and O was obtained using an Elementar Macro Cube elemental analyzer and all measurements were performed according to the EN15289-2011 standard protocol. To examine the surface morphology by scanning electron microscope (SEM), the stubs were coated using a JHC-1300 JEOL sputter coater, applying a dual-layer coating of gold of an approximate size of 4 to 6 nanometers in thickness. Subsequently, the surface morphology of both the secondary char and raw fructose was examined using the JEOL JSM-6010LA Scanning Electron Microscope (SEM). Python code was used to assist in counting the number of microspheres from the SEM images. ImageJ2 software by Fiji was used to measure the diameter of the microspheres. The assumptions for calculating the number and diameters can be found in the ESI file.† The functional groups were analyzed by a Bruker Alpha II Fourier-transform infrared analysis instrument accompanied by platinum attenuated total reflection (ATR) crystal with a resolution of 4 cm^−1^.

Further information regarding the detailed methodology, uncertainty and the limit of detection of all the conducted analyses, product fractions, and carbon fractions calculations can be found in Abdeldayem *et al.*^18^

### 2.2. Computational fluid dynamics model

#### 2.2.1. Geometry

The reactor was built according to its real dimensions using the COMSOL geometry building tool.^20^ The model geometry comprised a reactor vessel with an anchor-shaped impeller as illustrated in Fig. S-1.† The reactor's internal height (*H*) is 26.67 cm, while the diameter is 10.16 cm. The anchor-shaped impeller has a diameter (*d*) of 9.8 (cm). In addition to that, the vessel is equipped with a temperature probe of 1.15 cm diameter. After defining the geometry, the materials and their physical properties were selected for each domain using the COMSOL data library.

#### 2.2.2. Hydrodynamics solver

The rotating machinery module was used in COMSOL 6.2 to simulate the hydrodynamics. The equations of motion for single-phase flow are the continuity and momentum equations represented in eqn (2) and (3), respectively,^21^ and the hydraulic solver used to cover the range of flow conditions in the HTC reactor is the turbulent flow *k*–*ε* model with a frozen rotor study (steady state case for rotating machinery) using Reynolds-averaged-Navier–Stokes (RANS).2
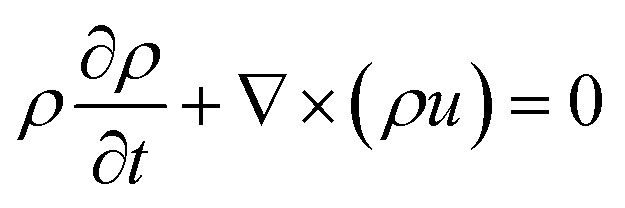
3
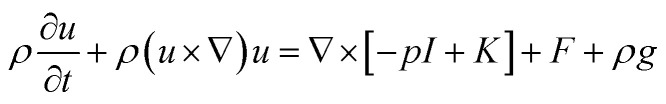
where *u* is the fluid velocity, *t* is the time, *p* is pressure, *I* is the unit tensor, *F* is the net of external forces, *k* is the viscous pressure tensor, *g* is the acceleration due to gravity.^22^

As the reactor operates in a closed system with no mass inflow or outflow during HTC, the boundary conditions were simplified by treating the system as a sealed domain. Reynolds-averaged-Navier–Stokes equations (RANS), which are time-averaged simplifications of Navier–Stokes equations, were used to solve the momentum and continuity conservation equations. The low memory requirements and adequate convergence rate allowed the *k*–*ε* RANS approach, which is common in CFD, to simulate the mean flow characteristics for turbulent flow conditions in stirred reactors.^17,23^ It is a well-established approach for industrial applications, particularly in cases with high Reynolds numbers.^23^ The *k*–*ε* model features two additional transport equations with two dependent variables: *k* is the turbulent kinetic energy (TKE), which is the average kinetic energy per unit mass associated with eddies in turbulent flow, reflecting the intensity of turbulence within the fluid,^24^*ε* is the turbulent dissipation rate illustrated in eqn (4) and (7).

The transport equation of *k* is4
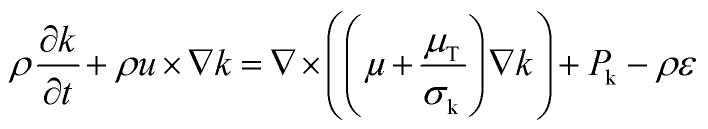
where the turbulent viscosity is,5
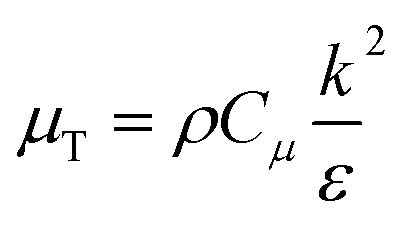
where the production term *P*_k_ is,6
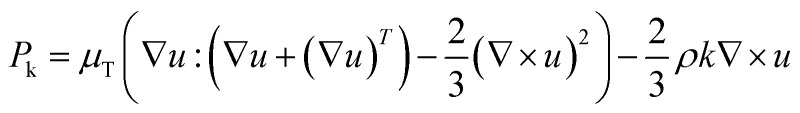
and the transport equation for *ε* is,7
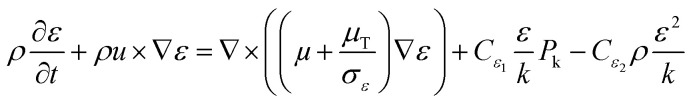
where *C*_*ε*_1__, *C*_*ε*_2__, *C*_*μ*_, and *σ*_*k*_ are model constants derived from experimental data^21,25,26^ mainly from Launder and Sharma.^26^

#### 2.2.3. Wall boundary

Wall functions were used to solve the flow near the walls, considering weakly compressible fluids. The stirrer shaft, impeller, and temperature probe were also considered to be a wall, *i.e.* rigid-body assumption. This assumption reflects the realistic behavior of the fluid adhering to solid surfaces (no-slip conditions), which is critical for accurately capturing velocity gradients near the reactor wall.

Additional information regarding the constraints and the wall functions can be found in COMSOL's documentation.^21,27^

#### 2.2.4. Mesh independence study

A sketch of the mesh can be found in Fig. S-1 in the ESI file.† The meshing of the reactor vessel can be summarized into 3 main components: (i) the fixed mesh where there is no movement of objects; (ii) the rotating moving mesh where the rotating domain rotates; and (iii) the deformed moving mesh where the free surface moves.

**Fig. 1:**
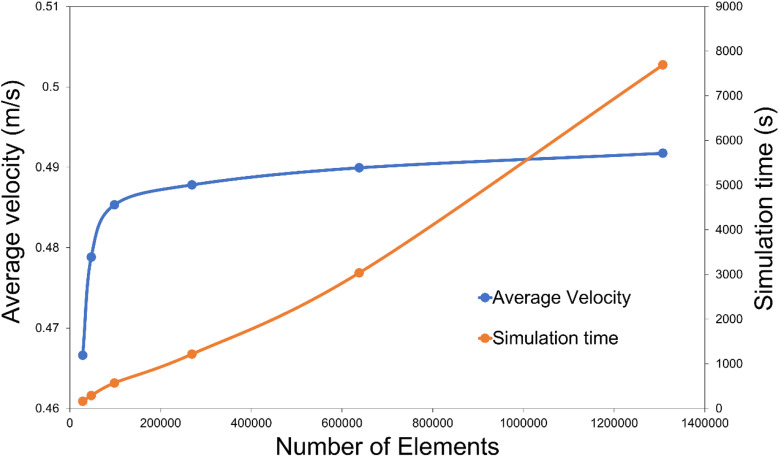
Mesh independence study.

The accuracy of the CFD solution relies mainly on the mesh quality. A mesh is considered independent if the simulation's accuracy does not significantly change when increasing the number of mesh elements.^28^ In the context of HTC scientific literature, there is no specific method for achieving a mesh-independent solution.^17^ Therefore, in this study, the average velocity of the fructose solution, which is the main dependent variable, was used to study the mesh independence. In this study, six different meshes were used. The mesh independence study was conducted at 200 rpm using the frozen rotor assumption (a particular steady state case) and stationary surface assumption. A virtual machine of 16 GB and 12 cores was used for this simulation. As shown in Fig. 1, it was noticed that the variation in average velocity tended to decrease and become stable above 100 000 elements. This was, therefore, the criterion to select the mesh applied in this study, which was composed of free tetrahedral nodes that varied in size between 0.27 and 2.22 cm.

## 3. Results and discussion

### 3.1. pH and mass balance of HTC products of fructose

**Fig. 2:**
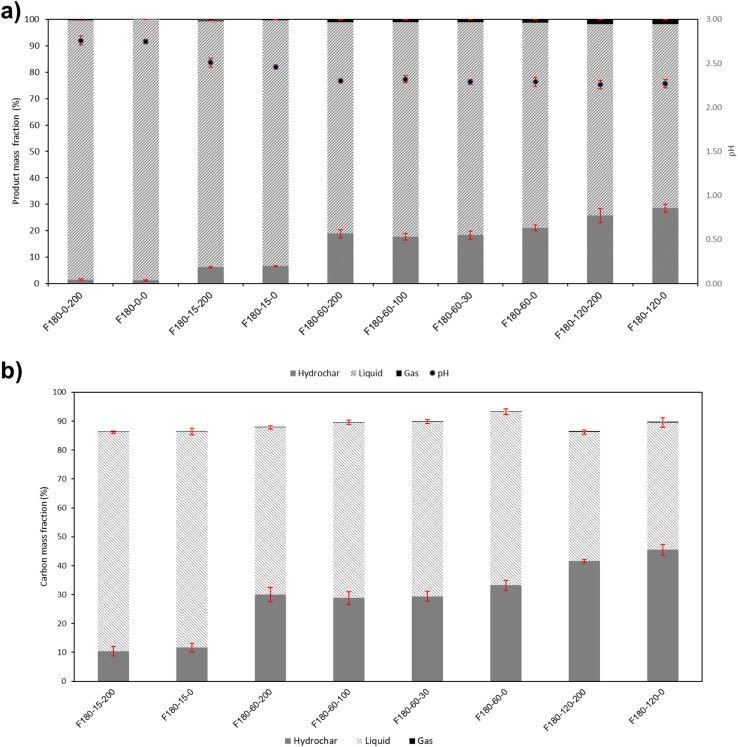
(a) pH and product mass fractions of HTC products of fructose at different residence times for stirred and unstirred conditions (b) carbon mass fraction at different residence times at stirred and unstirred conditions.

Fig. 2a shows the pH and product mass balance. Overall, the stirred and unstirred samples had very similar product mass fractions and pH at the studied conditions. Fig. 2a shows that there are minimal variations in the product distribution and pH measurements at 180 °C and 60 min of residence time for the four different stirring rates tested (0, 30, 100, and 200 rpm). This confirms that the stirring rate had no significant influence on the product distribution in the range tested, as found previously by Abdeldayem *et al.*^18,29^ with primary char using *Typha australis*, rejected apples, tomatoes, and digestate. There are two reasons that may contribute to the fact that stirring was found to have almost no influence on the studied properties. Firstly, water is a good medium for heat transfer and heat storage, and hence local peaks in temperature that might arise during HTC^30^ can be avoided. Secondly, the heating-up period was around 45 min, which may have been sufficient for natural convection to homogeneously transfer heat across the water medium in the reactor.^18^

At 0 min residence time (after finishing heating up), the solid mass fraction was around 2%, indicating that the hydrothermal reactions commenced during the heating-up period at a temperature lower than 180 °C, which is commonly known in literature as the lowest temperature of HTC. No similar observations were reported for fructose at these conditions, but similar findings were noted by Ischia *et al.*^3^ on initiating HTC reactions at lower temperatures using glucose as feedstock.

It was observed that upon increasing the residence time, the solid mass fraction tended to increase. The solid mass fraction for stirred and unstirred experiments was around 2%, 6%, 20%, and 29% for runs performed at 0 min, 15 min, 60 min, and 120 min, respectively. This indicates that in the early stages of HTC, fructose is almost completely dissolved in the water, and as the reaction progresses, secondary char is formed through condensation and polymerization reactions of the intermediates produced during the HTC process.^3,31^ The gas mass fraction was negligible in the studied experiments and reached a maximum of 2% at 120 min of residence time. The pH of water at the start of the experiment was 8.05 ± 0.02; however, it was noticed that it decreased over time, reaching around 2.3 at 120 min of residence time. This occurs mainly due to the formation of organic acids, such as formic acid, acetic acid, and levulinic acid, which are produced during the HTC reactions of fructose.^32^ These acids contribute to the catalysis of the HTC reactions of secondary char.^33^

### 3.2. Carbon balance of HTC products of fructose

The carbon balance has been conducted as shown in Fig. 2b. Overall, the carbon balance ranged between 87% and 94%, which agrees with previous studies conducted in the same reactor.^34,35^ The losses can primarily be attributed to the secondary char sticking to the reactor walls and potential losses during the solid, liquid, and gas extraction steps. Similar challenges related to secondary char losses from fructose were reported by Modugno *et al.*^36^ F180-0-200 and F180-0-0 were not considered in the carbon balance due to their limited mass available for analysis.

Similar to the solid mass fraction, the highest solid carbon fraction was obtained at 2 h. In contrast, the lowest solid carbon fraction was obtained at 15 min, indicating the impact of residence time on the formation of secondary char carbon fraction. The stirred samples exhibited a solid carbon fraction similar to the unstirred ones, indicating that the stirring rate had little influence on the solid carbon fraction. This is further confirmed by the fact that at a temperature of 180 °C and a residence time of 60 minutes, the carbon balance was very similar for all four stirring rates tested (0, 30, 100, and 200 rpm) as illustrated in Fig. 2b, again indicating that the stirring rate had no notable impact on the carbon balance.

Even though the solid carbon fraction in secondary char increased over time, as shown in Fig. 2b, it was noticed that the C, H, and O composition of the secondary char did not show significant differences, as illustrated in Table 2. Significant dehydration and decarboxylation occurred during the HTC of raw fructose, forming the secondary char, which is indicated by the lower H/C and O/C ratios of secondary char.^37^

**Table 2:** C, H, O, H/C, O/C compositions and ash content of secondary char

Run	C	H	O	H/C	O/C	Ash
Raw	42.13 ± 0.1	6.4 ± 0.0	51.21 ± 0.1	1.82	0.91	0.2 ± 0.1
F180-15-200	65.0 ± 0.3	4.5 ± 0.1	30.0 ± 0.3	0.82	0.35	0.2 ± 0.1
F180-15-0	66.6 ± 0.1	4.5 ± 0.1	28.8 ± 0.1	0.80	0.33	0.2 ± 0.1
F180-60-200	66.9 ± 0.1	4.1 ± 0.0	28.9 ± 0.1	0.73	0.32	0.1 ± 0.0
F180-60-100	67.6 ± 0.1	4.1 ± 0.1	28.3 ± 0.1	0.73	0.31	0.1 ± 0.0
F180-60-30	68.1 ± 0.1	4.7 ± 0.1	27.4 ± 0.1	0.77	0.30	0.1 ± 0.1
F180-60-0	66.4 ± 0.1	4.1 ± 0.0	29.4 ± 0.0	0.75	0.33	0.1 ± 0.1
F180-120-200	67.8 ± 0.1	4.0 ± 0.1	28.0 ± 0.1	0.71	0.31	0.1 ± 0.0
F180-120-0	67.1 ± 0.1	4.1 ± 0.0	28.7 ± 0.1	0.73	0.32	0.1 ± 0.0

The carbon content of secondary hydrochar was between 65 and 68%, which agrees with the theoretical carbon content of hydrochar (66.7%)^9^ and also is in agreement with previous studies.^9,38,39^Table 2 shows the ash content of the produced secondary char. Overall, the results were in the range of 0% to 0.2%, *i.e.*, almost no inorganic elements, as expected from fructose decomposition. In addition, the ash might originate from previous experiments (despite an extensive cleaning protocol) or experimental error in the proximate composition analysis.

### 3.3. Surface functional groups of secondary char

**Fig. 3:**
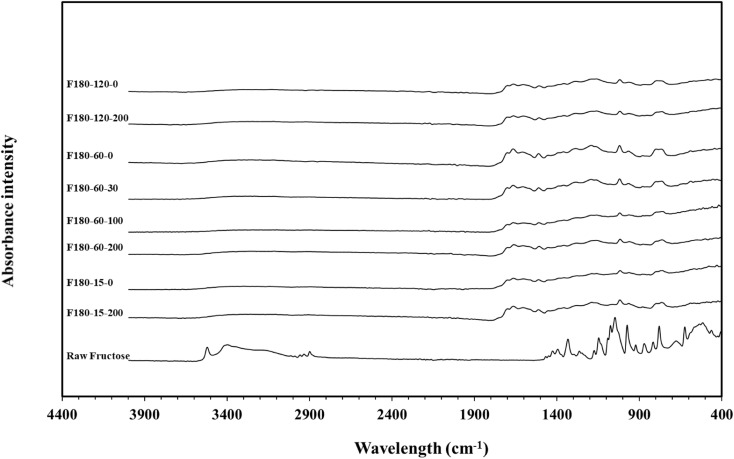
FTIR spectra for raw fructose and hydrochar produced at different residence times at stirred and unstirred conditions.

Fourier-Transform Infrared Spectroscopy (FTIR) spectra of raw fructose and the produced secondary char at different residence times in stirred and unstirred conditions are presented in Fig. 3. The raw fructose sample showed significantly more functional groups than the secondary char. The spectra of d-fructose can be divided into two main regions. As explained by Ibrahim *et al.*^40^ in their study on fructose, the first region from 1500–600 cm^−1^ represents C–O and C–C groups where the carbohydrates demonstrate their characteristic bands, while the second region mainly represents the C–H stretching obtained at 3000–2850 cm^−1^ and the –OH stretching vibrations between 3600 and 3100 cm^−1^.

The obtained FTIR spectra at different residence times and stirring conditions exhibit minimal differences across all the studied secondary char samples. This indicates that the residence time and stirring rate did not significantly influence the secondary char functional groups under the studied conditions. Secondary char is formed through polymerization and condensation reactions, where residence time primarily influences the product yield, particularly at low HTC temperatures, such as 180 °C.^3^ However, the properties of the resulting secondary char remain unchanged. Consequently, the secondary char spectra in Fig. 3 show no significant difference. The absence of any effect of stirring on the FTIR spectra may be attributed to the same reasons discussed in Section 3.1 regarding the absence of any impact on product mass balance. Water's high heat transfer efficiency prevents local temperature peaks,^30^ and the long heating-up time allows natural convection to distribute heat homogeneously throughout the reactor, reducing the need for stirring.^18^

Regarding surface functional groups of the obtained secondary char, the shoulder obtained at 1750 cm^−1^ indicated an undissociated carbonyl group.^41^ The vibrations obtained between 1700 and 1500 cm^−1^ are the characteristic stretching bands associated with the five-member heteroaromatic ring with double bonds. The bands observed at the 800–700 cm^−1^ range can be attributed to a pronounced hydrogen wag absorption of the five-membered ring containing the CH=CH unsubstituted group.^41^ Hence, the spectrum suggests that the secondary char is derived from hydroxymethylfurfural (HMF), as indicated by the distinctive five-member heteroaromatic rings.^41^ The HMF is identified as an intermediate compound produced through the dehydration of sugars under HTC conditions, which matches previous findings of researchers.^3,9,12^

### 3.4. Hydrodynamics of the HTC reactor

#### 3.4.1. Validation

**Fig. 4:**
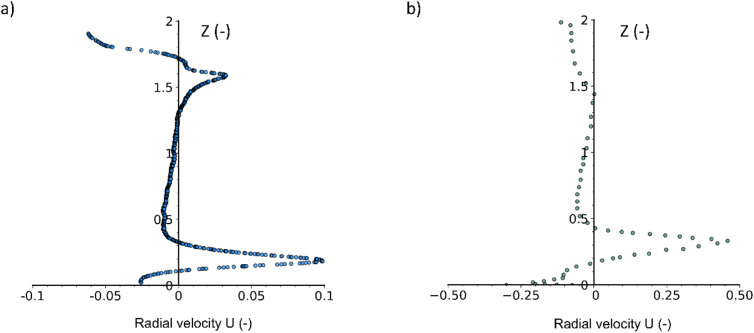
Axial profiles of radial velocity using classical anchor impeller (a) current study at 65 rpm (b) Karray *et al.*^42^ study at 60 rpm.

To ensure the validity of the developed model, the radial velocity across the axial profiles is compared with the validated model of Karray *et al.*^42^ at the anchor impeller's radius using water. The model developed by Karray *et al.*^42^ was validated with experimental power number data available in the literature. Fig. 4 illustrates the axial velocity profiles in the current study (Fig. 4a) and in Karray *et al.*^42^ study using a classical anchor (Fig. 4b). The *U* and *Z* resemble the dimensionless radial velocity and the dimensionless axial coordinate, respectively. Using a cylindrical reactor coordinate system, the radial velocity is illustrated where the radial velocity profiles are obtained in a stationary state (frozen rotor) at the tip of the impeller (*x* = 4.8 cm) in Fig. 4.

The main attributes of the graphs are similar, with a large peak observed in both Fig. 4a and b where it represents the horizontal blade of the anchor impeller. A second peak was observed in Fig. 4a but was much less pronounced in Fig. 4b. The second peak resembles a slight increase in the radial velocity at the top of the anchor blade of the impeller. It is important to notice that the velocity magnitudes in the two figures are different despite the fact that two stirring rates used were very close (60 and 65 rpm). This difference is due to the scale variation between the two setups. In our study, the vessel's inner and impeller diameters were 0.103 m and 0.098 m, respectively. In contrast, in the study by Karray *et al.*,^42^ the vessel's inner and impeller diameters were 0.3 m and 0.225 m. Even with geometrically similar reactors, these size differences result in higher velocities in the larger reactor when operated at the same stirring rate.^43^ Another reason for the slight difference in the second peak of the radial profile is that the radial coordinate of the impeller tip in Karray *et al.*^42^ study was at *r* = 0.5, whereas in the current study, it was at *r* = 0.96. This discrepancy could influence the radial velocity profile, as the clearance between the impeller and the reactor wall was greater in the study performed by Karray *et al.*^42^ compared to the current one.

#### 3.4.2. Hydrodynamics of the HTC reactor

In this study, the fructose solution is homogeneous; therefore, mixing is used to achieve homogeneous process conditions inside the reactor. The criterion for achieving that is based on Re, where a turbulent flow regime indicates homogeneous flow conditions inside the reactor.^17^

**Fig. 5:**
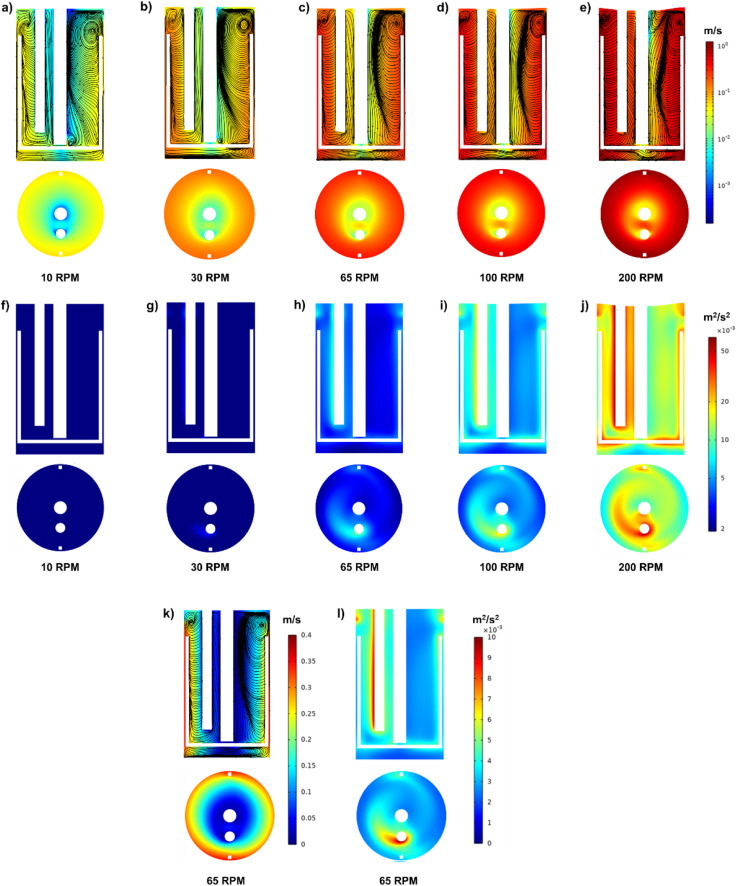
(a–e) Influence of stirring rate on the velocity fields inside the reactor (f–j) influence of stirring rate on the turbulence kinetic energy inside the reactor (k) influence of stirring rate at 65 rpm on the velocity fields and streamlines (l) influence of stirring rate at 65 rpm on the TKE.

The corresponding Re at 10, 30, 65, 100, and 200 rpm are shown in Table 3. At a low stirring rate of 10 rpm, the flow is in the transition regime, while at 65 rpm, the flow is at the start of the fully turbulent regime. Hence, the flow is then in the fully turbulent regime at 200 rpm. Fig. 5 shows the velocity fields at the different stirring rates. The cross-section velocity field is obtained at *z* = 10 cm, while the cut plane is obtained by cutting through the center of the reactor. Overall, it can be observed that at different stirring rates, different velocity fields and TKE of a different order of magnitude are obtained, with the highest values at 200 rpm and the lowest at 10 rpm, as illustrated in Fig. 5.

**Table 3:** Re and flow regimes at different stirring rates

Stirring rate (rpm)	Re	Flow regime	Average flow velocity (m s^−1^)	TKE (m^2^ s^−2^)
10	1.5 × 10^3^	Transition	0.03	0.000374
30	4.6 × 10^3^	Transition	0.08	0.001244
65	1.0 × 10^4^	Turbulent	0.17	0.003169
100	1.5 × 10^4^	Turbulent	0.26	0.005438
200	3.1 × 10^4^	Turbulent	0.53	0.013349

At 10 and 30 rpm, the average flow velocity in the vessel was 0.03 and 0.08 m s^−1^, respectively with the highest velocity obtained around the impeller as illustrated in Fig. 5a and b. At 65 rpm, the average flow velocity was 0.17 m s^−1^ in the reactor, and it was observed that it was higher at the anchor stirrer and the walls of the reactor vessel with a velocity of around 0.30 m s^−1^ as shown in Fig. 5c and k compared with the velocity in the center of the reactor (0.05 m s^−1^). Using a linear legend, Fig. 5k further elaborates on the different velocities at 65 rpm. Two main velocity zones are present inside the reactor: the first is between the stirrer and the reactor's walls, and the second is between the impeller and the stirrer axis. As expected, the velocity tends to decrease towards the stirrer shaft axis. Similar to previous studies with an anchor impeller, the velocity tends to be highest around the impeller.^44,45^Fig. 5d and e illustrate the velocity fields at 100 and 200 rpm. The average velocity inside the reactor vessel was around 0.26 and 0.53 m s^−1^ at 100 and 200 rpm, respectively. The velocity reached around 0.53 and 1.08 m s^−1^ between the wall and the impeller at 100 and 200 rpm, respectively. Similar to the findings at 65 rpm, the velocity tends to decrease towards the axis.

Fig. 5f–j show the TKE inside the reactor at 10, 30, 65, 100, and 200 rpm, and their corresponding values can be found in Table 3. These results further indicate the significant effect of increasing the stirring rate on the turbulence inside the reactor, confirming the results of Re shown in Table 3. Overall, it can be observed that the TKE is the highest around the stirrer and the temperature probe. Interestingly, the temperature probe works as a pier, leading to high turbulence shown as a tail towards the clockwise direction (as the rotation of the stirrer is clockwise), as illustrated in Fig. 5h–j. This might indicate that the temperature probe acted as a pier by disrupting the flow pattern and creating turbulence throughout the height of the reactor.

Fig. 5a–e further illustrates the velocity fields and streamlines inside the reactor vessel. The streamlines exhibit distinct circulation patterns on either side of the anchor stirrer and shaft. This suggests that the anchor stirrer is effectively generating rotational flow within the vessel.^46^ The flow velocity tends to be higher near the walls of the vessels as the anchor impeller tends to push the fluid flow outwards, which is similar to Karray *et al.*^42^ using water and an anchor stirrer. The streamlines between the stirrer and the walls occur as the anchor stirrer pushes the fluid towards the wall; then, it turns upward or downward along the walls, which will eventually lead to ascendant flow near the walls and descendant flow next to the shaft. At the top right and bottom left corners of the vessel, vortices exist, indicated by the streamlines. These vortices might occur due to the fluid being pushed against the vessel walls and recirculating. At the top of the vessel, this upward-moving fluid reaches the surface, where it encounters resistance (liquid–gas interface), causing the fluid to turn and recirculate back towards the center, forming a vortex.

### 3.5. Surface morphology of secondary char

#### 3.5.1. Effect of residence time on hydrochar at stirred and unstirred conditions

**Fig. 6:**
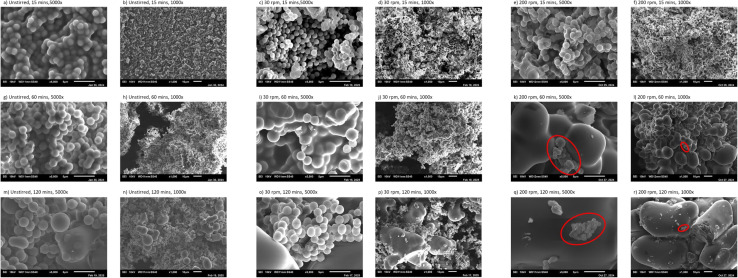
Morphology of various runs at different residence times under unstirred and stirred conditions: (a and b) 15 min, unstirred; (c and d) 15 min at 30 rpm; (e and f) 15 min at 200 rpm; (g and h) 60 min, unstirred; (i and j) 60 min at 30 rpm; (k and l) 60 min at 200 rpm; (m and n) 120 min, unstirred; (o and p) 120 min at 30 rpm; (q and r) 120 min at 200 rpm.

Upon looking at the unstirred samples in Fig. 6a and g, it can be observed that uniform, smooth, and spherical microspheres were obtained with an average diameter of around 1 μm, which is consistent with previous studies using fructose (without additives) in unstirred conditions,^8,9^ as well as other carbohydrates.^3,13^ For these unstirred experiments (Fig. 6a and g), it was noted that the sizes of the microspheres were 1.56 ± 0.15 and 1.61 ± 0.16 μm, respectively, for the runs conducted at 30 and 60 min. Even though uniform spherical microspheres were still observed in the SEM at a 120 min of residence time, larger particles were also present, which were not observed at shorter residence times; hence, they were further investigated. The unstirred experiment conducted at 120 min of residence time and exhibited in Fig. 6m and n indicate the existence of both smooth uniform spheres and larger aggregates. The small spheres had a size of 2.22 ± 0.44 μm, while the larger aggregates had a size of 10.66 ± 4.56 μm. This indicates that particle size increases as residence time increases even during unstirred conditions, similar to the findings of Jung *et al.*^9,12^ However, this is only observed in the run conducted at 120 min.

The surface morphology of hydrochar produced at 30 rpm is shown in Fig. 6c, i and o for residence times of 15, 60, and 120 minutes, respectively. As illustrated in Fig. 6c, the majority of particles are uniform spherical microspheres with a diameter of 1.25 ± 0.13 μm. At a residence time of 60 minutes and 30 rpm, the spheres tend to agglomerate and aggregate, forming larger clusters while the original spherical particles remain visible, as shown in Fig. 6i. At 120 minutes (Fig. 6p), larger particles with varying sizes can be observed, reaching up to 66 μm in length, while the smaller particles have a diameter of 2.19 ± 0.17 μm.

The obtained microspheres at stirred conditions of 200 rpm are shown in Fig. 6e, k and q at 5000× magnification. Similar to Fig. 6a and c, Fig. 6e shows a significant amount of microspheres; most of which are uniformly spherical and very similar but with a slightly deformed morphology. Increasing the residence time while stirring, increases the aggregated microspheres' size, as illustrated in Fig. 6k and l, and Fig. 6q and r. Even though microspheres of 1–3 μm can still be found on the top of the aggregates and also in other locations in the studied samples, in this study we focused on the observed large aggregates of various sizes and irregular shapes. The sizes of the aggregates were around 5–30 μm and 55–70 μm at 60 and 120 min, respectively.

Overall, this section highlights that stirring accelerates the aggregation of particles into larger aggregates. Moreover, particles tend to aggregate and form larger structures as the residence time increases, as illustrated by the findings under the selected conditions.

#### 3.5.2. Effect of stirring rate at different locations in the HTC reactor

**Fig. 7:**
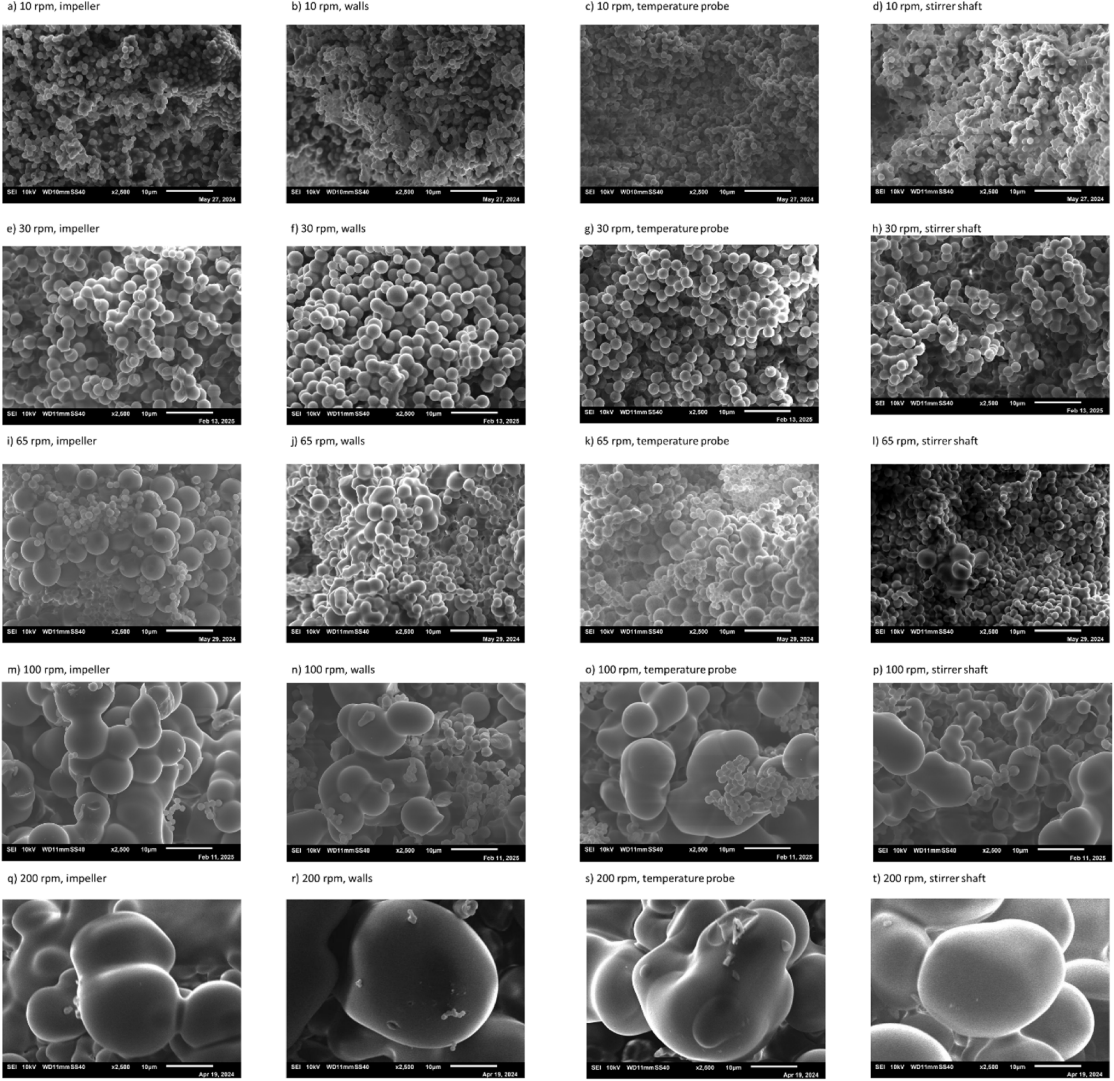
Morphology of secondary char produced at 60 min of residence time at (a–d) 10 rpm, (e–h) 30 rpm, (i–l) 65 rpm, (m–p) 100 rpm, and (q–t) 200 rpm, observed at the impeller, walls, temperature probe, and stirrer shaft.

Upon looking at the secondary char obtained at 1 h and 10 rpm Fig. 7a–d, it was noticed that the majority of the microspheres had a spherical and uniform morphology. The average diameter of microspheres varied between 1.21 and 1.28 μm at all the studied locations, as shown in Table 4. The spherical uniform morphology and the absence of bigger microspheres illustrate that the stirring rate is not high enough to improve the aggregation of secondary char, which can be justified by the low turbulence indicated by the low Re and TKE illustrated in Table 3 and Fig. 5a and f, respectively.

**Table 4:** Number and average diameter of the microspheres at different locations of the reactor at 1 h of residence time

Stirring rate (rpm)	Location	Number of particles	Minimum size (μm)	Maximum size (μm)	Average diameter (μm)
10	Impeller	1108	0.91	1.57	1.21 ± 0.15
	Wall	822	0.96	1.51	1.22 ± 0.14
	Temperature probe	1210	1.00	1.55	1.26 ± 0.11
	Stirrer shaft	971	1.09	1.63	1.28 ± 0.12
30	Impeller	314	1.81	3.03	2.73 ± 0.31
	Wall	395	1.83	4.75	2.69 ± 0.48
	Temperature probe	531	1.98	2.61	2.36 ± 0.13
	Stirrer shaft	460	2.01	2.58	2.32 ± 0.11
65	Impeller	344 + (64)^a^	0.96	5.89	1.59 ± 0.21 (5.26 ± 0.33)
	Wall	280 + (48)^a^	1.28	6.82	1.81 ± 0.27 (4.12 ± 1.00)
	Temperature probe	351 + (69)^a^	1.06	5.29	1.84 ± 0.39 (3.96 ± 0.52)
	Stirrer shaft	703 + (9)^a^	1.11	4.62	1.57 ± 0.24p (3.69 ± 0.73)
100	Impeller	28 + (50)^a^	1.14	12.73	1.14 ± 0.19 (8.52 ± 1.82)
	Wall	246 + (36)^a^	0.93	23.45	1.72 ± 0.42 (10.79 ± 6.35)
	Temperature probe	189 + (23)^a^	2.17	24.85	1.67 ± 0.30 (13.65 ± 4.79)
	Stirrer shaft	126 + (30)^a^	1.40	17.07	2.11 ± 0.39 (11.18 ± 3.95)
200	Impeller	—	—	—	31.31
	Wall	—	—	—	37.30
	Temperature probe	—	—	—	36.85
	Stirrer shaft	—	—	—	31.06

a The numbers in brackets for 65 rpm demonstrate the number and average size of big particles in the figures.

The secondary char obtained at 1 h and 30 rpm can be found in Fig. 7e–h. The majority of the observed microspheres at all locations exhibited uniform spherical morphology. The average diameter of the obtained microspheres was 2.32–2.73 μm at the studied location, as shown in Table 4.

The experimental runs conducted at 65 rpm Fig. 7i–l reveal interesting findings. Overall, all samples had larger particles (up to 6.82 μm) compared with the samples obtained at 10 and 30 rpm as illustrated in Table 4, which can be attributed to the fact that 65 rpm corresponds to the onset of the fully turbulent regime, achieved at Re > 10^4^.^19^ The larger particles can be further explained due to the turbulent regime and the fact that the TKE and velocity field are 1 order of magnitude higher than in the case of stirring at 10 rpm, as shown in Fig. 5c and h (65 rpm) compared to Fig. 5a and f (10 rpm).

At 65 rpm, small microspheres and larger aggregates were observed. In proportion, large aggregates were much less abundant than small microspheres in the studied samples. The average large aggregates at these locations were 5.26 ± 0.33, 4.12 ± 1.00, and 3.96 ± 0.52 μm for the impeller, walls, temperature probe, and stirrer shaft, respectively as shown in Table 4. It is important to mention that the majority of the particles were small microspheres of 0.96–2.76 μm that can be found in all runs. The existence of large aggregated microspheres can be attributed to the fully turbulent regime, as illustrated by the Re. The large aggregated microsphere concentration at the impeller, walls, and temperature probe coincides with the fact that the velocity fields and turbulence are highest around these regions of the reactor compared to the middle of the reactor around the stirrer shaft, as demonstrated by the hydrodynamic study in Fig. 5c and h and further illustrated by Fig. 5k and l which illustrates the velocity fields and turbulence using a linear legend instead of a logarithmic legend. A large number of small particles of an average diameter of 1.20–2.12 μm were found at the stirrer shaft illustrated in Fig. 7l, while having a few large aggregated microspheres ranging between 2.42 and 4.63 μm.

The observed samples at 100 rpm showed small microspheres in addition to larger aggregates as shown in Fig. 7m–p. The aggregates varied in size from 8.52 to 13.65 μm at the studied different locations, as illustrated in Table 4. The growth pattern and aggregation of particles continued at 200 rpm, where large aggregates were observed that varied in size between 31 and 37 μm, as illustrated in Table 4 and Fig. 7q–t. This is almost 1 magnitude larger than the microspheres obtained at 0, 10, and 65 rpm.

### 3.6. Discussion

#### 3.6.1. Theories of secondary char growth mechanism in literature

There are two main theories that have been proposed regarding the formation and growth of secondary char. Both theories agree on the formation of HMF from sugars. In the early days of secondary char formation studies, it was commonly assumed that secondary char microspheres formed through a nucleation and growth mechanism, as described by the LaMer model.^47–49^ When the concentration of aromatic clusters in the solution reaches the supersaturation point, burst nucleation occurs. The formed nuclei then grow further as more molecules from the solution diffuse to their surface.^47–49^

Zhang *et al.*^31^ introduced another theory (aggregation model) based on hydrophobic ripening. They state that the polycondensation and polymerization of HMF lead to the removal of hydroxyl terminals and continuous growth of the molecular cluster that gradually becomes hydrophobic, producing primary nanoparticles. The primary nanoparticles develop into micro-sized carbonaceous uniform spheres in an isotropic environment by aggregating primary nanoparticles into secondary char through intermolecular dehydration and the interaction of functional terminals, which minimizes the system's surface-free energy.

In their study, Zhang *et al.*^31^ demonstrated that LaMer model theory may not be fully correct in the context of secondary char formation due to the following reasons: (i) the LaMer model suggests that nucleation and growth occur when the concentration of the feedstock exceeds a critical level. However, in their study, the results indicated that carbonaceous spheres could form even at low concentrations of HMF, far below the solubility limit, which contradicts the oversaturation requirement of the LaMer model (ii) the LaMer model relies on a burst nucleation event where numerous particles form simultaneously once the critical concentration is reached. However, the experimental evidence indicates that the formation of carbonaceous spheres does not involve this event. Instead, the process is more gradual, with continuous formation and growth of the particles, which deviates from the predictions of the LaMer model (iii) in the LaMer model, embryonic particles are considered unstable until a critical size is reached. However, the study found that the polymeric embryos (formed from HMF) are stable entities that do not require such a critical threshold to precipitate. This stability supports the hydrophobic ripening theory, where the primary nanoparticles form and grow without the need for oversaturation, and (iv) the formation kinetics of the carbon spheres do not conform with the LaMer model profile.

Another study conducted by Jung *et al.*^9^ further supported the theory developed by Zhang *et al.*^31^ and further demonstrated that LaMer model theory may not be the most accurate mechanism for the secondary char formation context. Their findings indicated that significant particle growth occurs after the concentration of HMF has decreased to low concentrations, indicating that the primary mechanism of particle growth is not continuous nucleation from the monomer but rather coalescence of existing particles. Moreover, their SEM images indicated that secondary char particles started to merge and aggregate into larger particles over time. This process of particle merging supports the hydrophobic ripening theory, as it illustrates that the particles grow not by adding more monomeric HMF from the solution but by coalescing with other particles.

#### 3.6.2. Influence of stirring rate on secondary char formation and growth

The probability of collision and interactions of HMF monomers determines the formation of primary nanoparticles.^31^ This is followed by the aggregation of the primary nanoparticles, forming larger microsphere particles (secondary char) through aggregation as time increases. Therefore, a slight increase in secondary char production is observed with longer residence times, as shown in Section 3.1.

Even though the secondary growth mechanism developed by Zhang *et al.*^31^ was performed in unstirred conditions, the theory can still be extended to stirred conditions. From the results of the current study, it is evident from the surface morphology shown in Fig. 7 that there is a stirring rate threshold at which particles show significant aggregation and an increase in size. The threshold appears to occur around 65 rpm, corresponding to the start of fully turbulent flow, as illustrated by the aggregated particles in Fig. 7. Beyond this point, increasing the stirring rate enhances particle aggregation and growth, as observed in Fig. 7, whereby particles at 200 rpm were nearly an order of magnitude larger than those obtained at 65 rpm.

From the product mass and carbon balance in Fig. 2a and b, it can be observed that stirred conditions did not increase the solid mass fractions obtained compared with unstirred conditions at all studied residence times, indicating that stirring did not increase the formation rate of secondary char. However, the surface morphology of secondary char obtained in stirred conditions in Fig. 6 (at different residence times) and in Fig. 7 (at different stirring rates) indicates that stirring has significantly increased the aggregation of smaller particles. The irregular aggregated shape of secondary char obtained in Fig. 6 and 7 in stirred experiments might be attributed to stirring, which introduced more kinetic energy into the system, which is clearly observed by the TKE at 10 rpm (0.000374 m^2^ s^−2^) and 200 rpm (0.005438 m^2^ s^−2^) in Fig. 5f and j. Hence, stirring leads to an acceleration in the coalescence and aggregation of particles, leading to non-uniform growth of spherical particles and promoting a more irregular morphology. This interpretation is aligned with the velocity fields and TKE illustrated in Fig. 5, where the highest velocity and TKE were achieved at the highest stirring rate (200 rpm, see Fig. 5e and j), which is consistent with the largest microsphere particle sizes achieved at 1 h and 2 h at 200 rpm as shown in Fig. 6k and q.

#### 3.6.3. Mechanism of formation of secondary char on the solid walls of the reactor

It was also observed that the secondary char obtained in stirred experiments tends to form a thick layer on the reactor walls and stirrer, with negligible secondary char dispersed in the liquid phase, similar to the observations of Modugno.^36^ Fig. S-2 in the ESI file† illustrates the thick layer on the stirrer, temperature probe, and the reactor's wall. This might occur due to the velocity fields inside the reactor vessel, whereby the initially produced solid nanoparticles of secondary char follow the streamlines shown in Fig. 5a–e, which might result in their deposition on the temperature probe, stirrer shaft, impeller, or walls due to the no-slip flow conditions, forming a thin layer. Over time, more solid nanoparticles are produced and follow the same streamlines, leading to their agglomeration and aggregation on the thin layer, forming hydrophobic carbonaceous microspheres. Over time, more microspheres are formed, leading to a thick layer by the end of the experiment. Based on the intensity of the velocity field at each region and the streamlines, the accumulation density of the deposited microspheres will vary.

These findings are relevant for scaling up industrial HTC systems for secondary char collection and production. By employing adequate stirring, thin baffles or piers could be strategically placed inside the reactor to promote turbulence and secondary char accumulation, facilitating collection at the end of the experiment.

### 3.7. Future research

#### 3.7.1. Future research and perspectives

To further advance our understanding of the influence of stirring on HTC and secondary char formation, future research should focus on employing more advanced quantitative methods that use dynamic image analysis, such as a Camsizer, for particle counting and shape analysis of secondary char which should provide additional insights into particle size distribution and aggregation behaviour in HTC. Additionally, investigating a wider range of stirring rates, especially in the turbulent regime, will allow more accurate identification of the threshold above which stirring has a significant impact on particle size. This will, in turn support a more in-depth explanation of the underlying mechanisms related to secondary char formation. Besides that, expanding the scope of the research to include other sugars will also be crucial in verifying the aggregation theory, particularly the concept of hydrophobic ripening with a wider range of substrates. The majority of existing studies have focused on secondary char formation using simple sugar feedstocks, primarily monosaccharides. It is therefore recommended to investigate secondary char formation in both stirred and unstirred conditions using more complex feedstocks, such as polysaccharides (*e.g.*, cellulose or hemicellulose), and potentially lignocellulosic biomass at a later stage. This would enhance our understanding of particle aggregation mechanisms for secondary char formation and allow for broader generalization of the findings across different process conditions and feedstocks. Besides that, it is essential to have more research coupling hydrodynamic and experimental approaches for secondary char formation, particularly integrating secondary char aggregation mechanisms in a multiphysics or CFD model. Finally, in the current study, the influence of stirring on the formation of carbon dots was not investigated due to experimental constraints. However, it is also recommended to further explore the impact of stirring on carbon dots.

#### 3.7.2. Potential applications of secondary char and opportunities for scaling up

Secondary char is characterized by its spherical morphology, high carbon content, and tunable surface properties, and has emerged as a promising material in advanced applications.^3^ For example, secondary char has the potential to act as an electrode material in energy storage devices such as supercapacitors and batteries. Its high carbon content, electrical conductivity, and specific capacitance make it ideal for electrochemical applications. For instance, nitrogen-doped secondary char enhances electron transport and stability, yielding specific capacitances that improve energy storage performance.^50^

According to the results of Ischia *et al.*,^3^ secondary char exhibits photoluminescence, which is the spontaneous emission of light upon photoexcitation. According to the author's knowledge, secondary char photoluminescence properties have not been utilized in previous applications; therefore, its potential to be used in advanced applications should be explored.

For the successful scaling up, industrialization, and commercialization of hydrochar, particularly secondary char, it is essential to focus on applications in advanced, high-value industries. Secondary char holds significant potential for such uses; however, relying solely on model compounds like fructose or glucose may not be economically viable. These model compounds are valuable feedstocks for research studies under controlled conditions and provide critical insights into the mechanisms of secondary char formation. Findings based on model compounds can then be applied to more practical and cost-effective feedstocks, such as fruit waste, which is rich in sugars and readily available. By leveraging waste feedstocks, the production of hydrochar and secondary char could become both economically feasible and sustainable, paving the way for broader adoption in industrial applications.

## 4. Conclusions

This study addressed the gap in understanding the effects of stirring on various properties of secondary char produced from HTC, using fructose as a model compound. Experimental results demonstrated that stirring does not influence the pH, mass, or carbon balance of secondary char under the conditions tested. However, stirring significantly impacted the morphology of the microspheres, with larger particle aggregates forming at stirring rates > 65 rpm (Re > 10^4^) and at residence times > 60 min. The study identified a stirring rate threshold corresponding to the onset of the turbulent flow regime, above which particle aggregation was more pronounced, likely due to the development of fully turbulent flow. Additionally, the hydrodynamic model revealed that at the onset of the turbulent flow regime (65 rpm), regions of high velocities and turbulence corresponded to areas where large aggregates of secondary char formed in the reactor, indicating that these hydrodynamic factors probably play a critical role in particle aggregation. Finally, a mechanism explaining the deposition of thick layers of secondary char on the reactor walls and the stirrer in stirred conditions is proposed, driven by the velocity streamlines within the reactor.

## Abbreviations

### List of symbols

ρ
: Fluid density
u
: Fluid velocity
t
: Time
P
: Hydrostatic pressure
I
: Unit tensor
F
: Net of external forces
K
: Viscous pressure tensor
g
: Acceleration due to gravity
d
: Diameter of the impeller
N
: Impeller's rotational frequency
μ
: Dynamic viscosity
k
: Turbulent kinetic energy (TKE)
ε
: Turbulent dissipation rate
*C*_*ε*_1__, *C*_*ε*_2__, *C*_*μ*_, and *σ*_*k*_: Turbulent model constants from experimental data
*n*_i_ *τ*
: Normal stress
σ
: Surface tension coefficient'

### List of acronyms

ATR: Attenuated total reflection
BET: Brunauer–Emmett–Teller
CFD: Computational fluid dynamics
DOC: Dissolved organic carbon
HMF: Hydroxymethylfurfural
HTC: Hydrothermal carbonization
SEM: Scanning electron microscope
TKE: Turbulence kinetic energy
RANS: Reynolds-averaged-Navier–Stokes
Re: Reynolds number
MCR: Modular compact rheometer

## Data availability

Data will be made available on request.

## Conflicts of interest

There are no conflicts to declare.

## Supplementary Material

RA-015-D4RA08995B-s001
