# Supplementary material for: An experimental and numerical investigation of secondary char formation in hydrothermal carbonization: revealing morphological changes via hydrodynamics
Source: RSC Adv. 2025 Apr 23;15(16):12723–38. doi: 10.1039/d4ra08995b (PMC12015937; doi:10.1039/d4ra08995b)
Supplement: RA-015-D4RA08995B-s001 [file RA-015-D4RA08995B-s001.pdf]

Supplementary Information (SI) for RSC Advances.
This journal is © The Royal Society of Chemistry 2025

An experimental and numerical investigation of secondary char formation in hydrothermal carbonization: Revealing morphological changes via hydrodynamics

Omar M. Abdeldayem[1,2,*], Capucine Dupont[1], David Ferras[1], Maria Kennedy [1,2]

[1] Department of Water Supply, Sanitation and Environmental Engineering, IHE Delft Institute for Water Education, Westvest 7, 2611AX Delft, the Netherlands

[2] Department of Water Management, Faculty of Civil Engineering and Geosciences, Delft University of Technology, Stevinweg 1, 2628 CN Delft, the Netherlands

***Correspondence to:**

Omar M. Abdeldayem

Email: o.m.h.m.abdeldayem@tudelft.nl

Assumptions for measuring particle size

1) If the boundaries of merged particles are observable, then they were counted with the number of observed merged particles

2) If the boundaries of merged particles are not observable, then they are counted as one

3) If the particle is slightly hidden underneath some other particles, then it is counted, but its length is not measured

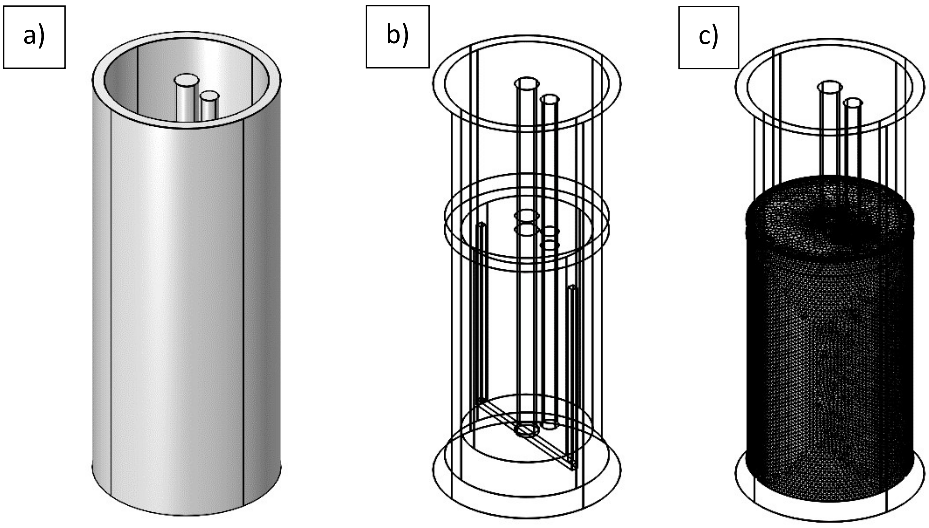

Figure 1 Mesh sketch of the reactor

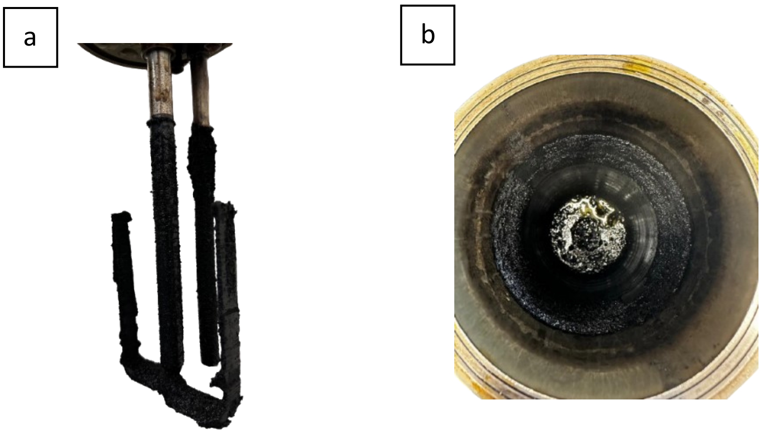

Figure 2 a) Hydrochar on the temperature probe, stirrer shaft, and impeller b) hydrochar on the reactor's vessels walls
